# Increasing self‐efficacy and knowledge in carer training: Hispanic versus Caucasian

**DOI:** 10.1002/nop2.376

**Published:** 2019-09-27

**Authors:** Leisa Easom, Ke Wang, Gayle Alston

**Affiliations:** ^1^ Georgia Southwestern State University Americus GA USA

**Keywords:** carers, dementia, self‐efficacy

## Abstract

**Aim:**

Nurses are teachers to their patients and need to know best practices for diverse families living with dementia. Little is known about Hispanic beliefs around dementia knowledge and self‐efficacy that may have an impact on the learning situation.

**Design:**

A pre‐/postresearch design was used in this intervention study with a baseline assessment of dementia knowledge and caregiver self‐efficacy and a reassessment at training completion.

**Methods:**

Investigation of education training with two caregiver groups caring for persons with dementia: Caucasian and Hispanic. Convenience sample consisted of 567 Caucasians and 104 Hispanic dementia caregivers. Groups received training in their primary language accompanied by a training book (Dealing with Dementia Guide) also in the primary language.

**Results:**

Dementia knowledge and caregiver self‐efficacy increased in both groups with the Hispanic group demonstrating significantly greater increase in self‐efficacy. The Caucasian group had a significantly greater increase in the dementia knowledge compared with the Hispanic group.

## 1 INTRODUCTION AND BACKGROUND

Minority populations are often disadvantaged in their abilities to access and understand health care in their community (Kagan, 2017). The language barrier is often cited as the most common challenge with 51.3% of Hispanics reporting the need for a translator when seeking health care in the United States (Duran, 2012). Effective nurse–patient communication is critical for the process of healthcare delivery as language barriers may result in lack of understanding of medication purposes and possible side effects, healthcare instructions and follow‐up. A language barrier represents an important challenge for Hispanic access to health care, especially for adults who are cognitively impaired (Vega, Cabrera, Wygant, Velez‐Ortiz, & Counts, 2017). Contributing to this health barrier issue is the growing number of older Hispanics living with cognitive impairment in the United States (Alzheimer's Association, 2019). Alzheimer's disease affects twelve per cent of older adults in the Hispanic or Latino population, the highest proportion among different ethnic groups in the United States (Alzheimer's Association, 2019). The terms, Hispanic and Latino, are used interchangeably in the literature. For our research, the term Hispanic is used to denote all those who speak Spanish as a primary language in the United States.

Hispanics constitute the third fastest growing population in the United States. This population is projected to increase to 119 million in 2060, representing a 115 per cent increase from 2014. By 2060, 29 per cent (almost one‐third) of the USA is projected to be Hispanic (U.S. Census Bureau, 2015). The older Hispanic community continues to age with the number of Hispanics over the age of 65 doubling by year 2030 (Vega et al., 2017). Older Hispanics are one and one‐half times as likely to have Alzheimer's disease or other dementias as older Caucasians (Alzheimer's Association, 2019). Complicating this issue is the language barrier, with only 40% of Hispanics 69 years or older being fluent in English. Central to any communication is the ability to understand what is being taught and shared. This language barrier limits access to resources and understanding of training and education that is available.

Contributing to the stress of a language barrier is also the lack of knowledge about dementia and the belief that one can be confident of one's ability to provide good care of an individual with dementia. Increasing one's knowledge is affirming, but the confidence to manage a situation is critical. Self‐efficacy is the belief of one capabilities to assess and carry out the proper courses of action to manage a situation. One's self‐efficacy beliefs affect one's coping resources, degree of effort and persistence to keep on trying even when the outcome appears bleak. Individuals with lower self‐efficacy lack the confidence to persist and may give up without much effort. Those individuals with higher self‐efficacy have a “can do” attitude and a strong belief in their capabilities. Both knowledge and self‐efficacy are integral components to success in caring for another, especially in dementia care (Depp et al., 2005).

Additionally, cultural backgrounds can be linked with behaviour and can predict behaviour and choices. In the United States, the traditional notion of the melting pot symbolization of cultural integration has been replaced by the salad bowl metaphor, indicating that the various groups retain the cultural values and habits from the country of origin (Beniflah & Chatterjee, 2015). In regard to caregiving, there is little known about Hispanic beliefs around dementia caregiving and practices that may have an impact on the carer learning situation.

Overall, little is known about the impact on carer groups from different cultural in their self‐efficacy and dementia knowledge through exposure to a structured dementia training workshop using printed materials in the primary language of the participant. The present intervention study was to assess and evaluate dementia knowledge and self‐efficacy in two cultural groups (Hispanic and Caucasian) prior to training participation and at training end.

## 2 METHODS

### 2.1 Design, setting and sample

This intervention study used a pre‐ and postresearch design with baseline assessment of carers occurring prior to the workshop and reassessment at workshop end. The final convenience sample of workshop attendees were 567 Caucasian, non‐Hispanic family carers and 104 Hispanic family carers. The Caucasian carers were recruited from their communities through service providers and community marketing by the trainers. Hispanic carers were recruited through the Hispanic Ministries in the United Methodist Churches in their respective towns. Inclusion criterion was that the carer must currently be caring for someone living with dementia. Most Hispanic carers (79.1%) were female and less than 60 years of age while the Caucasian carers (71.3%) were female and more than 60 years of age. Most of the carers from both groups were from urban areas and had been caregiving for <5 years. Table 1 provides carer demographic characteristics.

**Table 1:** Carer demographic characteristics

Variable category	Caucasian, non‐Hispanic carer *N* (%)	Hispanic carer *N* (%)	Association between demographic variable and carer type *p* value
Gender			
Male	110 (20.2)	30 (30.9)	<.001
Female	434 (79.8)	67 (69.1)	
Age (years)			
<60	160 (28.7)	68 (79.1)	.023
≥60	398 (71.3)	18 (20.9)	
Resident location			
Urban	478 (85.7)	92 (97.9)	.004
Rural	80 (14.3)	2 (2.1)	
Employment status			
Employed full‐time	125 (22.3)	67 (69.8)	<.001
Employed part‐time	45 (8.1)	20 (20.8)	
Primary family carer	51 (9.1)	2 (2.1)	
Retired	303 (54.1)	2 (2.1)	
Other	36 (6.4)	5 (5.2)	
Education			
1–11 years	9 (1.6)	61 (68.5)	<.001
High school grad	85 (15.3)	19 (21.4)	
Some college	166 (29.9)	5 (5.6)	
College grad	179 (32.2)	4 (4.5)	
Masters	99 (17.8)	0	
Doctorate	18 (3.2)	0	
Length of caregiving (years)			
<5	394 (60.4)	71 (79.8)	<.001
5–10	142 (29.1)	17 (19.1)	
>10	51 (10.5)	1 (1.1)	
Relationship to care recipient			
Spouse	188 (35.7)	7 (7.7)	<.001
Unmarried partner	4 (0.8)	7 (7.7)	
Child	212 (40.3)	17 (18.7)	
Parent	30 (5.7)	14 (15.4)	
Other relative	78 (14.8)	29 (31.8)	
Non‐related	14 (2.7)	17 (18.7)	

A successful training workshop requires a structured educational plan with appropriate printed materials to facilitate learning and allows active participation. All trainers (Hispanic and Caucasian) used the same training programme—Dealing with Dementia Guide. The Dealing with Dementia Guide for Carers was originally developed by the Rosalynn Carter Institute for Caregiving (RCI) as part of the evidence‐based carer support programme RCI REACH (Resources Enhancing Alzheimer's Carer Health). RCI REACH is a powerful multi‐component carer support programme delivered one on one by a Certified Carer Coach over a 6‐month period. One hundred per cent of carers who participated in the original RCI REACH programme rated the Guide as very helpful in their evaluation of the programme (Easom, Alston, & Latini, 2017). The guide is a tabulated, indexed comprehensive reference guide for carers. To facilitate easy reading and understanding, the entire guide is available in English and Spanish, written at a fifth‐grade reading level, and uses large font and bold headings to guide the reader's eye to the information they seek.

There are six sections of the guide:

- Introduction: illustrates a deep understanding of the carer experience and mindset in a supportive and encouraging tone;
- Understanding Dementia: basic information on how dementia affects the brain, the four most prevalent types of dementia and the basic stages of dementia;
- General Caregiving Tips: chapters on safety, medication management, building care teams, creating a dementia‐friendly environment, etc.;
- Dealing with Behavioral Issues: demonstrates a nine‐step problem‐solving model with applications to dementia behaviours and chapters on specific behaviours such as agitation, bathing, wandering and incontinence;
- Taking Better Care of Yourself: discusses the importance of self‐care, six stress management strategies, asking for and receiving help, carer depression and carer grief, etc.;
- Resources: lists the types of services available with checklists to facilitate selection of service providers and lists national resources with contact information.

The Dealing with Dementia (DWD) 4‐hr training workshop focuses on teaching carers how to use this vast resource guide. The training is conducted in a classroom‐style workshop. Carers are led through the DWD guide from front to back, highlighting topics that are known quagmires for carers and pointing out the topics they may want to return to review later. Every participant receives a guide to take home. The workshop's underlying theme is to connect with, applaud and empower both family and professional carers. The examples and tips shared during the workshop demonstrate a deep understanding of the caregiving experience and offer suggestions for managing daily challenges. Book topics include depression, dementia behaviours such as aggression and communication challenges, self‐care, stress management, seeking and accepting help, creating dementia‐friendly lifestyles and problem‐solving.

The workshop training leaders had various educational backgrounds, but all received the same instruction on how to conduct the 4‐hr dementia education from the Rosalynn Carter Institute for Caregiving (RCI) staff (authors of the Guide). These leaders (41 Caucasian with English as first language and 1 Hispanic with Spanish as first language) completed a 1‐day Trainer Class in which they were instructed in the background of the DWD Guide, the theme of the workshop (not only to inform but also to applaud and lift up carers) and operational issues around delivery and expectations from RCI. To complete the training and demonstrate mastery of skill required for a DWD Master Trainer, each individual delivered a mock workshop with at least three observers critiquing performance. The mock workshop allowed the new trainee to practice the delivery of content required and share real‐life examples. With approval from the observers, the workshop training leader was prepared to conduct the community training workshops.

### 2.2 Ethical approval

This study was approved (IRB#15‐009) by the Institutional Review Board of the Georgia Southwestern State University. Each participant also signed a consent form available in their primary language.

### 2.3 Intervention

The primary objective of the study, which was based on Knowles Adult Learning Theory (Knowles, 1984), was to measure and observe two different cultural groups (Hispanic and Caucasian) responses (similarities and/or differences) in regard to changes in self‐efficacy and dementia knowledge when participating in a 4‐hr dementia training workshop. According to the theory, adults are most interested in learning about a topic which relates directly to their lives (Knowles, Swanson, & Holton, 2005). Each of the carers in the study was active carers of persons with dementia and was eager to participate and learn.

Over a period of 1 year, a total of 10 four‐hour workshops were held with Hispanic carers and 25 four‐hour workshops were held with Caucasian carers. Data were collected at the beginning of the workshop (before training started) and at training end with reliable and valid tools (Revised Scale for Caregiving Self‐Efficacy and the Alzheimer's Disease Knowledge Scale) and through observation by the workshop leaders.

### 2.4 Measures

In addition to obtaining demographic information on each training workshop participant, two valid and reliable scales were used to capture data related to self‐efficacy and knowledge of Alzheimer's Disease/Dementia. The Revised Scale for Caregiving Self‐Efficacy (RSCSE) is a 15‐item measure. It contains three subscales (5 items for each subscale)—Self‐efficacy for Obtaining Respite, Self‐efficacy for Responding to Disruptive Patient Behaviors and Self‐efficacy for Controlling Upsetting Thoughts about caregiving. The scale rated the degree of confidence from 0%–100%, where a 0% confidence meant that the carer could not do it at all, a 50% confidence meant that if the carer gave it his or her best effort, chances were about 50–50 that the carer could perform the activity and a 100% confidence meant the carer is certain he or she can do it. A total score was calculated by summing the responses, ranging from 0–500. Scales can be used separately or together. Past studies reported an alpha coefficients of 0.080 (reliability) or greater for each of the three of the subscales (Gilliam & Steffen, 2006). This study used the Self‐Efficacy for Responding to Disruptive Patient Behaviors scale (five items).

The Alzheimer's Disease Knowledge Scale (ADKS) was used to assess the carers' knowledge about Alzheimer's Disease. The 30‐item, true/false scale addressed seven content domains that encompassed the breadth of information: risk factors, assessment and diagnosis, symptoms, course, life impact, caregiving and treatment and management. A total score ranged from 0–30. Studies show that the ADKS has adequate reliability (test–retest correlation = 0.81 and internal consistency alpha coefficient = 0.71) and validity (content, predictive, concurrent and convergent; Carpenter, Balsis, Otilingam, Hanson, & Gatz, 2009; El‐masry, Elwasify, & Khafagy, 2018). The ADKS has also been translated into multiple languages, including Spanish.

### 2.5 Analysis

Statistical analysis was performed using SPSS for Windows version 24. Paired‐sample *t* tests examined differences within the mean from baseline to programme completion for the variables of self‐efficacy and Alzheimer's disease knowledge. Frequency distributions were performed to analyse descriptive data. Statistical significance was considered at *p* < .05.

## 3 RESULTS

Both groups had statistically significant increases in self‐efficacy (SE) and Alzheimer's knowledge (ADKS) from baseline to programme end assessment (all *p* <.001, Table 2). Specifically, the mean SE score of Caucasian non‐Hispanic carers was 357.05 at baseline and 389.05 at the follow‐up, with a mean increase of 32.00; the mean SE score of Hispanic carers was 377.26 at baseline and 441.30 at the follow‐up, with a mean increase of 64.04. The mean ADKS score of Caucasian non‐Hispanic carers was 24.71 at baseline and 28.35 at the follow‐up, with a mean increase of 3.64; and the mean ADKS score of Hispanic carers was 16.73 at baseline and 19.59 at the follow‐up, with a mean increase of 2.86 (Table 2).

**Table 2:** Assessment scale score comparison

Assessment scale score	Caucasian non‐Hispanic carer mean (*SD*)	Hispanic carer mean (*SD*)	Association between scale score and carer type *p* value
Pre_SE	357.05 (101.64)	377.26 (61.74)	.007
Post_SE	389.05 (88.06)	441.30 (65.26)	<.001
Diff (Post‐Pre)_SE	32.00 (75.40)*	64.04 (76.06)*	<.001
Pre_ADKS	24.71 (3.27)	16.73 (2.66)	<.001
Post_ADKS	28.35 (2.02)	19.59 (2.96)	<.001
Diff (Post‐Pre)_ADKS	3.64 (3.28)*	2.86 (4.06)*	.063

Abbreviations: ADKS, Alzheimer's Disease Knowledge Scale; SE, Self‐Efficacy.

* Indicates *p* <.001, the statistical significance of SE and ADKS change from baseline to programme end assessment.

The Hispanic family carers tended to be younger with 79.1% of Hispanic carers under 60 years old and 28.7% of Caucasian non‐Hispanic carers under 60 years old. Additionally, 90.6% of Hispanic family carers were employed, in either full‐time (69.8%) or part‐time (20.8%) positions, while 54.1% of Caucasian non‐Hispanic family carers were not employed (retired).

Hispanic carers reported less education than Caucasian non‐Hispanic carers: 68.5% of Hispanic carers received 11 or less years of education and 10.1% received higher education (college). Comparatively, 1.6% of Caucasian non‐Hispanic carers received 11 or less years of education and 83.1% received higher education (college and higher). Last, most (82.5%) of Caucasian non‐Hispanic family carers were the immediate relatives of the care recipients, composed by spouses/partners, parents and children. In Caucasian families, the carer was the child caring for a parent, whereas the Hispanic carer was more often a non‐immediate relative of the family member.

## 4 DISCUSSION

The purpose of this intervention study conducted a comparison of the results of training with two carer groups caring for persons with dementia: Caucasian (primary English speaking) and Hispanic (primary Spanish speaking). Healthcare providers working with these populations can use the lessons learned from this comparison as they incorporate healthcare training for individuals caring for someone with dementia. Conflicts may occur between nurses' cultural values and care practices in dementia care settings; thus, there is a need for ongoing education to decrease the possibility of such conflicts (Kang, Moyle, & Venturato, 2011).

The Dealing with Dementia (DWD) training workshop has led to statistically significant increases on the SE and ADKS of carers for both Hispanic and Caucasian groups. The net increase in the positive DWD effect on SE of carers was significantly different between the two groups: the SE increase in Hispanic carers was significantly greater than that of the Caucasian non‐Hispanic carers. This is an interesting result as the education levels of the Hispanic group were lower than the Caucasian non‐Hispanic group. Earlier research (Gallagher‐Thompson, Arean, Rivera, & Thompson, 2001) identified that barriers among Hispanic carers to access to care services included the carers' language proficiency and limited education. Perhaps having the training materials translated into Spanish and at a fifth‐grade reading level encouraged learning and knowledge uptake.

The positive effect on ADKS occurred similarly between the two groups. In other words, the increases (pre–post) in ADKS score were quite close between the groups. However, the ADKS scores (both baseline and after follow‐up) of the Caucasian carers were significantly higher than those of the Hispanic carers. This may be explained by the lower educational level. Researchers have found that Hispanics providing care to a family member with Alzheimer's disease and related dementias face numerous language, literacy and cultural barriers to accessing and housing health services (Weitzman, Neal, Chen, & Levkoff, 2008). Healthcare providers working with the Hispanic population need to have printed materials in Spanish and at a lower reading level for easy understandability. Other researchers (Hirata & Harvath, 2017) found that residents with dementia experienced stress which transferred to carer worker stress and staff turnover. Perhaps increasing carer knowledge of dementia may translate into lower stress for residents and carer worker retention as well.

Researchers have also found that training and intervention programmes with Hispanic carers were slow, labour intensive and required twice the amount of time allotted initially (Gallagher‐Thompson et al., 2001; Llanque & Enriquez, 2012). In the current study, we found that the Hispanic carer group did require more trainer attention and responded positively to more interaction. The Hispanic carer group voiced that they would like to do a craft and take it home to the care recipient as a way to include the recipient in the training. The cultural influence of collectivism may explain these findings. Collectivism relates to altruistic motivations and the desire to strengthen social ties according to Finkelstein (2010). Hispanic culture embraces collectivism with beliefs that the group is more important than the individual, whereas the Caucasian culture values individualism. The healthcare provider must be aware that cultural values and family variables may play a statistically significant role in adherence to the healthcare regime and plan for more time and family inclusion when planning health training.

Some limitations of the current study should be noted. The different backgrounds of the trainers and the difficulty assuring consistent delivery of training in the field are limitations in this study. The limited geographical area may influence the generalizability of the results. Future studies should include a more diverse groups of cultural backgrounds to participate in the training.

## 5 CONCLUSIONS

With the prediction that Hispanics will occupy one‐third of the population of the United States in future years, strategies to reach and teach carers of this group are important. The current study findings indicated that best practices for healthcare instruction include that printed materials translated into the primary language are important for the teaching message to be understood. Allowing more time and incorporating more group interaction within the teaching time were anecdotal findings that can have a positive impact on the learning experience. These practices contributed to increased self‐efficacy (confidence) and increased dementia knowledge levels for the Hispanic carers in this study.

## CONFLICT OF INTEREST

No Conflict of Interest.
